# Using a Three-Dimensional Collagen Matrix to Deliver Respiratory Progenitor Cells to Decellularized Trachea *In Vivo*

**DOI:** 10.1089/ten.tec.2018.0241

**Published:** 2019-02-14

**Authors:** Nick J.I. Hamilton, Robert E. Hynds, Kate H.C. Gowers, Angela Tait, Colin R. Butler, Colin Hopper, Alan J. Burns, Martin A. Birchall, Mark Lowdell, Sam M. Janes

**Affiliations:** ^1^ Lungs for Living Research Centre, UCL Respiratory, Division of Medicine, University College London, London, United Kingdom.; ^2^ UCL Ear Institute, The Royal National Throat Nose and Ear Hospital, London, United Kingdom.; ^3^ Department of Biochemical Engineering, University College London, London, United Kingdom.; ^4^ Maxillofacial Surgery, Eastman Dental Institute, London, United Kingdom.; ^5^ Stem Cell and Regenerative Medicine, Birth Defects Research Centre, UCL Great Ormond Institute of Child Health, London, United Kingdom.; ^6^ Institute of Immunity and Transplantation, Centre for Cell, Gene and Tissue Therapeutics, Royal Free Hospital, London, United Kingdom.

**Keywords:** tissue engineering, regenerative medicine, mucosa, airway reconstruction, tracheal transplantation, epithelial cells

## Abstract

Airway epithelial cells are a key barrier to inhaled toxicants, pollutants, and infectious agents. During severe epithelial injury and in transplantation settings, it would be desirable to rapidly reintroduce a functional epithelium through the engraftment of exogenous cells or by promoting host regeneration. However, the three-dimensional surface of the airway and the requirement to maintain airflow using stents, which cause mechanical shear at the tracheal surface, are challenges. In this study, we investigate a graft composed of human airway epithelial stem/progenitor cells and lung fibroblasts embedded within a supportive collagen matrix, which we propose might be more resistant to mechanical shear than cells exposed directly to the lumen surface. Cells were combined in a collagen I hydrogel before it was dehydrated into a mechanically stable sheet using RAFT^™^ absorbers. After 48 h, KRT5+ spheroids formed and bromodeoxyuridine staining indicated active proliferation. To test whether epithelial cell- and fibroblast-containing grafts were able to successfully engraft on a section of trachea *in vivo*, they were implanted onto revascularized, decellularized tracheal scaffolds in a rabbit model. After 1 week, constructs had engrafted with signs of revascularization and keratin-positive cells were found throughout the scaffold. Although the long-term fate of these cells *in vivo* remains uncertain, we envisage that this strategy could improve host epithelial repair and/or contribute directly to mucosal regeneration.

**Impact Statement:**

This article describes a method for engrafting epithelial progenitor cells to a revascularized scaffold in a protective and supportive collagen-rich environment. This method has the potential to overcome two key limitations of existing grafting techniques as epithelial cells are protected from mechanical shear and the relatively hypoxic phase that occurs while grafts revascularize, offering the opportunity to provide epithelial cells to decellularized allografts at the point of implantation. Advances in this area will improve the safety and efficacy of bioengineered organ transplantation.

## Introduction

Current strategies to regenerate lost or damaged upper airway epithelium are suboptimal.^1^ Conventional methods employ split skin grafts that, due to a high turnover of epidermal cells, can result in cell sloughing into the airway and infection.^2^ Skin grafts also retain hair follicles leading to irritation and the retention of secretions. Mucosa can be harvested from the lining of the inner cheek, but the supply of tissue is more limited and covering surface areas of greater than 5 × 2 cm^2^ is challenging.^3^ A method that restores damaged epithelium back to its native form is therefore desirable and would provide novel treatment options for patients who have complex upper airway mucosal disease or require reconstruction following cancer resection.^4,5^ The ability to deliver a new airway epithelial layer to a tracheal allograft would also overcome one of the limitations of bioengineered tracheal transplantation.^6,7^

**FIG. 1.**
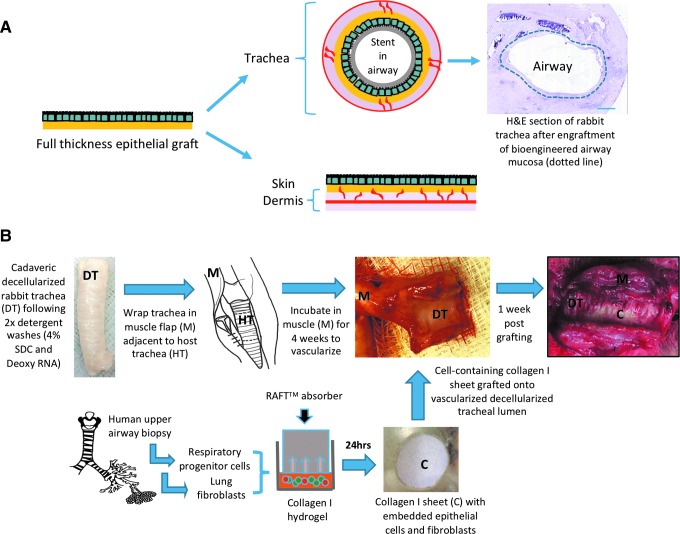
A strategy for regenerating upper airway mucosa using cell-containing collagen I-based scaffolds. **(A)** Full-thickness grafts are phenotypically comparable to native mucosa with an epithelial layer overlying a submucosal layer. Full-thickness grafts can be harvested from healthy areas of skin or buccal mucosa or they can be bioengineered by seeding epithelial cells onto a biological scaffold. Full-thickness grafting has been used for skin restoration where it is implanted on the surface of a flat, well-vascularized graft bed. In contrast, the implantation of a full-thickness graft within the airway requires grafts to be manipulated onto a cylindrical surface and a stent to hold it in place, damaging the epithelial layer. The vascular supply to the luminal surface of the airway is also focused around the intercartilaginous septa and the underlying tissue is therefore less vascular than in skin, prolonging revascularization of the epithelium. Methods have not yet been developed in which the epithelial layer of a bioengineered respiratory mucosal full-thickness graft (*dotted line*) survives grafting within the rabbit trachea. **(B)** In our method, cultured human airway basal cells and lung fibroblasts were expanded *in vitro*. These cells were then suspended into a collagen I gel and dehydrated into a mechanically stable graft using RAFT^™^ absorbers. This generated a sheet that could be easily manipulated. These cell-containing scaffolds were engrafted onto a section of decellularized rabbit trachea that had been prevascularized in a muscle flap. Color images are available online.

Including epithelial cells in full-thickness airway grafts is complicated by their complex three-dimensional (3D) structure and delayed revascularization of the epithelial surface. Furthermore, the surface epithelial layer of a full-thickness graft is exposed to mechanical shear during manipulation into the airway by the surgeon and through friction with the overlying stent that holds grafts in place and maintains a patent airway (Fig. 1A). In clinical tracheal transplantation cases, these factors have led to the view that transplanted epithelial cells are unlikely to have contributed to epithelial restoration.^6,7^ A number of factors, including the delayed and incomplete epithelialization following transplantation, suggest this and the eventual regeneration of epithelium over the scaffolds has most likely been mediated by ingrowth of host epithelial cells.

Two possible approaches might improve epithelial regeneration in such cases. One is the direct application of epithelial stem cells to a prepared wound bed, with the intention that cells engraft and restore the epithelial layer.^8^ This strategy has been successfully employed to regenerate the entire skin surface using epidermal cells applied on a fibrin carrier in a child with a severe blistering skin disorder.^9^ However, direct translation of this technique into the airway is problematic as the cells are still exposed on the surface of the fibrin carrier and therefore liable to sloughing during manipulation and stenting. A second approach is to apply epithelial cells with the intention of expediting endogenous regeneration. This approach also has precedent in epidermal and oral mucosal epithelial cell therapies where cells can act as a biological dressing that stimulates reepithelialization from the adjacent healthy native epithelia.^10^

Stem cell-based 3D organoid cultures expand epithelia *in vitro* and their use in transplantation contexts is beginning to be explored.^11^ Transplantation of colonic organoid-derived cell suspensions in a murine model of acute colitis demonstrated that stem cells can engraft and contribute to histologically normal epithelium.^12,13^ In the lung, cells from human pluripotent cell-derived organoids can contribute to repair in a tracheal injury model.^14^ However, these studies involve the use of cell suspensions at the point of delivery, which has been inefficient in airway preclinical models and in clinical applications.^15^ Another approach has seen organoid-derived cells seeded onto scaffolds for transplantation: human extrahepatic cholangiocytes seeded on polyglycolic acid scaffolds contributed to gallbladder reconstruction in a murine model,^16^ and murine or human intestinal organoid-derived cells could be transplanted into the mouse omentum on a synthetic matrix.^17^

In this study, we investigated the transplantation of cultured human airway basal stem/progenitor cell^18^ cultures in 3D collagen scaffolds. Airway basal cells can be grown as 3D spheroids in Matrigel to generate “tracheospheres.”^19^ As Matrigel is not appropriate for clinical transplantation due to its murine sarcoma origin, we investigated whether a collagen matrix functioned similarly in an *in vitro* airway differentiation assay. Next, by embedding culture-expanded basal cells,^20–22^ along with lung fibroblasts, within a collagen gel and then dehydrating it, we generated a mechanically stable, cell-containing collagen I-based sheet. As proof of concept, we demonstrate successful grafting of these scaffolds in an immunosuppressed *in vivo* rabbit model. Such scaffolds might protect cells from environmental shear and provide a supportive microenvironment to help cells withstand the relatively hypoxic phase immediately after grafting. If regeneration is not mediated by long-term engraftment of these cells, they might also stimulate host epithelial regeneration.

## Methods

### Primary cell isolation and expansion

Tissue and biopsy collection were approved by the UK Research and Ethics Council (REC references 06/Q0505 and 11/LO/1522). Primary airway cells were isolated from routine airway endoscopy procedures and lung resections. All samples were transported on ice in a medium containing streptomycin (50 μg/mL), penicillin (50 IU/mL), and amphotericin B (1 μg/mL). Epithelial cells were isolated by explant expansion or by first digesting tissue overnight in 0.15% (w/v) pronase in DMEM at 4°C on a rotator. DMEM containing 10% fetal bovine serum (FBS) was then used to neutralize the pronase solution at a ratio of 2:1. Samples were then centrifuged at 300 *g* for 5 min to form a cell pellet before resuspension in epithelial growth medium containing 5 μM ROCK inhibitor Y-27632 (Enzo Life Sciences, Exeter, United Kingdom) and seeding into flasks containing a mitomycin C-treated 3T3-J2 feeder layer as previously described.^20,23^

Primary human lung fibroblasts (a kind gift from Prof. Robin McAnulty; University College London, United Kingdom) were maintained in DMEM (Gibco, Hemel Hempstead, United Kingdom) containing 10% FBS and were used no later than passage 10.^24^

### Collagen graft preparation

Rat tail collagen at a concentration of 2 mg/mL (type I, #60-30-810; First Link, Wolverhampton, United Kingdom) was mixed with Minimal Eagle's Medium 10 × (Gibco; #21430) in a ratio of 8:1 over ice. The mix was neutralized with 5 M NaOH until it turned pink in color. The solution was left on ice for 30 min to remove any bubbles. Primary human airway epithelial cells and primary human lung fibroblasts were then seeded into the gel in DMEM (Gibco; #21969) at a concentration of 1 × 10^6^ cells and 1 × 10^4^ per mL of gel mix, respectively.

1.3 mL or 0.25 mL of gel mix with cells was then transferred to wells of a 24-well or 96-well plate, respectively, and incubated at 37°C for 15 min to allow the gel to set. Custom-made RAFT^™^ absorbers (Lonza, Slough, United Kingdom; #016-1R33/016-1R32) were inserted over the gels within the plates. The absorbers were left for 15 min at room temperature to absorb water from the collagen gel and, in doing so, increase the collagen concentration of the final construct. After 15 min, the absorbers were removed and the epithelial cell culture medium was added to the collagen grafts on the bottom of the wells. The cell-containing collagen scaffolds were then returned to the incubator and cultured in epithelial cell culture medium for 1 week with daily media change after which they were cultured in differentiation medium. The protocol for collagen construct fabrication is based on previous work by colleagues who have used this approach to provide a limbal stem cell delivery method.^25^ Collagen constructs containing fibroblasts only were fabricated by seeding human lung fibroblasts at 4 × 10^4^ per mL of collagen type I, prepared using the methodology described above and maintained in DMEM containing 10% FBS.

### Optical coherence tomography

Scaffold thickness was measured using optical coherence tomography (Vivosight, Maidstone, United Kingdom; #TP1301). Full-thickness scans were taken at three points on each of four scaffolds derived from starting collagen concentrations of 1 or 2 mg/mL. ImageJ (version 2,0.0-rc-43/1.52i) was used to measure thickness.

### Three-dimensional tracheosphere culture

Three-dimensional tracheospheres were cultured to provide a positive control for spheroid formation and for the expression of airway differentiation-associated markers. Ultra-low attachment 96-well plates (Corning, Flintshire, United Kingdom; #CLS3474) were coated with 30 μL 25% growth factor-reduced Matrigel (Sigma-Aldrich, Dorset, United Kingdom; #E6909) on ice and this was allowed to gel at 37°C for 20 min.^23^ 2500 airway epithelial cells in 65 μL 5% growth factor-reduced Matrigel were then seeded on top of the 25% Matrigel layer. Tracheosphere medium consisted of a 50% DMEM, 50% Bronchial Epithelial Growth Medium (BEGM) mix with all BEGM bullet kits added except gentamicin/amphotericin, retinoic acid, and triiodothyronine. All-trans retinoic acid was added at a final concentration of 100 nM immediately before use. Twenty-five microliters additional tracheosphere medium (with retinoic acid) was added to each of the wells on day 1, 3, 8, and 14. On day 21, the well contents were removed into 15 mL Falcon tubes on ice, centrifuged at 400 *g* for 5 min, and then fixed in 4% paraformaldehyde for 30 min. Tracheospheres were then washed in phosphate-buffered saline (PBS) and resuspended in Histogel (Thermo Fisher, Hemel Hempstead, United Kingdom; #HG-4000-012) before processing and embedding in paraffin wax.

### Histology

Samples for histology were washed twice in PBS and placed in 10% neutral buffered formalin (NBF) for 1 h. Samples were processed in cassettes through an alcohol gradient using a Leica TP 1050 tissue processor. Samples were then embedded in paraffin wax. Paraffin-embedded samples were sectioned at 5 μm thickness using a microtome. Samples were mounted using a Sakura Coveraid automatic cover-slipping machine with Tissue-Tek cover slipping film (Sakura, Alphen aan den Rijn, The Netherlands). Deparaffinization for immunofluorescence staining was carried out using xylene and rehydration through a decreasing ethanol gradient using an automated system (Tissue-Tek DRS, Sakura). Haematoxylin and eosin (H&E) staining was performed using the same system and images were obtained using a NanoZoomer 2.0-HT system.

### Immunofluorescence

For whole-mount, top-down images, cell-containing collagen scaffolds were washed twice in PBS and then fixed in 10% NBF for 1 h. Scaffolds were then washed twice in PBS for 5 min each and blocked with 10% FBS in PBS for 2 h with the addition of Triton X-100 when staining spheroid cultures. Primary antibodies (BrdU [Bio-Rad, Watford, United Kingdom; OBT0030G], pan-keratin [Abcam, Cambridge, United Kingdom; ab9377], keratin 5 [Abcam; ab17130], MUC5AC [Sigma-Aldrich; M5293], ACT [Sigma-Aldrich; T6793], collagen IV [Abcam; ab6311], fibronectin [Thermo Scientific; PA1-84601], laminin alpha 4 [Abcam; ab69634]) were applied to the scaffold in block solution and incubated overnight at 4°C. After three 5-min washes in PBS, species-specific secondary antibodies were applied at 1:500 in block solution at room temperature for 1 h. Scaffolds were washed twice in PBS and 4′,6-diamidino-2-phenylindole (DAPI, 5 mg/mL stock, 1:10,000 in PBS; Life Technologies, Hemel Hempstead, United Kingdom) was applied. Samples were washed with PBS and coverslips applied before image acquisition using a Zeiss 700 confocal microscope. For bromodeoxyuridine (BrdU) staining, 100 μM BrdU (Sigma-Aldrich; #B5002) solution in medium was added to each scaffold and incubated for 12 h before fixation.

For paraffin sections, slides were dewaxed using an automated process (DRS-601; Sakura, Thatcham, United Kingdom). Slides were washed in distilled water and incubated in block solution for 2 h. Primary antibodies were applied in block solution and incubated at 4°C overnight. Following three PBS washes, species-specific secondary antibodies (AlexaFluor; Thermo Fisher) were applied in block solution at a concentration of 1:500 for 1 h at room temperature. Slides were washed twice more in PBS and stained with DAPI (5 mg/mL stock, 1:10,000 in PBS; Life Technologies) for 10 min. Following a final PBS wash step, slides were coverslipped using ImmuMount (Thermo Fisher). Slides were imaged on a Zeiss 700 confocal microscope.

### Cell viability assay

To assess epithelial cell viability within collagen scaffolds, we used a Live/Dead^™^ cell viability assay (Life Technologies; #L3224) according to the manufacturer's instructions. Collagen grafts containing only epithelial cells were fabricated as described above. After 24 h, the grafts were washed twice with PBS within the well. The assay reagents containing calcein AM and ethidium homodimer-1 were added and the scaffolds incubated for 45 min at 37°C. Following incubation, the scaffolds were removed and placed on a glass slide and coverslipped with ImmuMount. Scaffolds were imaged using a Zeiss 700 confocal microscope. Cell counts of live (green) cells and dead cells (red) were recorded from two images per scaffold by manual counting.

### Chick chorioallantoic membrane assay

Fertilized White Leghorn chicken eggs were supplied by Henry Stewart and Co. (Norfolk, United Kingdom). On embryonic day 3 (E3), eggs were cleaned with 70% ethanol and 3–4 mL of albumin was removed to create an air pocket in the apex of the shell. A 2 × 2 cm window was cut over the apex to reveal the embryo and chorioallantoic membrane (CAM) blood vessels. Windows were sealed with adhesive tape and the eggs incubated for a further 5 days. On E8, the CAM was reexposed and minutia pins used to disrupt several of the smaller blood vessels. Two square millimeter sections of acellular collagen graft and decellularized dermis were placed gently onto each CAM. The window was resealed and the eggs further incubated until E14, where they were imaged with a Leica Zoom 2000 stereomicroscope with camera adaptor (Magnifi^™^; Arcturus Labs, Kansas) to assess vascularization.

### Engraftment of collagen sheets onto a decellularized section of rabbit trachea

Tracheas were retrieved from terminated New Zealand white rabbits (Envigo; Huntingdon, United Kingdom) and stripped of all fascia before washing thrice in PBS containing penicillin and streptomycin (1 × ; Gibco) over 24 h. A two-cycle enzymatic detergent decellularization protocol was then employed to remove all cellular material from the trachea^26^ ahead of sterilization using ionizing irradiation.

For implantation experiments, male New Zealand white rabbits 9–10 weeks of age weighed 2.5–3 kg on arrival. General anesthesia was induced using 0.5 mL/kg ketamine intramuscularly and 0.2 mL/kg of xylazine and maintained with sevoflurane. All drugs were supplied by the National Veterinary Services (Stoke-on-Trent, United Kingdom). Five centimeter sections of decellularized trachea were preimplanted in a lateral thoracic muscle flap raised from the chest and tunneled under the cervical skin into the neck to revascularize the decellularized trachea for 4 weeks ahead of collagen scaffold engraftment (Fig. 1B). The day before implantation, the constructs were prepared as described above and maintained in culture for 24 h ahead of grafting onto the section of revascularized trachea. At the point of implantation, the cervical incision was reopened and the scaffold identified in the muscle wrap. The trachealis was identified and opened by incision directly through muscle. The tracheal rings were assessed for evidence of vascularization and structural integrity, and signs of infection. The epithelial collagen graft was then placed on the vascularized trachea and a stent placed over the graft to keep it in place. The trachea was then sutured closed around the stent and the trachea reimplanted with the cervical neck wound, which was then closed. Animals were terminated after 1 week and scaffolds were retrieved and prepared for histology by fixation in 10% NBF for 24 h ahead of processing as described above.

Immunosuppression of rabbits was required to reduce the probability of acute rejection of the transplanted human cells. Dexamethasone was administered at a dose of 2 mg/kg thrice daily beginning 24 h before grafting. Tacrolimus (Cambridge Bioscience, Cambridge, United Kingdom) was also administered subcutaneously at 0.3 mg/kg for 3 consecutive days, beginning 7 days before the day of surgery and then on alternate days until the end of the experiment.

## Results

### Characterization of the collagen I-based scaffold.

A starting gel volume of 1.3 mL in 24-well plates resulted in a scaffold surface area of 1.9 cm^2^, while a starting gel volume of 0.25 mL in 96-well plates resulted in a scaffold surface area of 0.32 cm^2^ (Table 1). Scaffold thickness varied with starting concentration of collagen gel: a 2 mg/mL concentration resulted in a mean thickness of 158 μm (standard deviation [SD] 23.15), whereas a 1 mg/mL collagen concentration lead to a mean thickness of 103.6 μm (SD 24.74).

**Table 1.** Characterization of Collagen-I Scaffolds

*Concentration of collagen (mg/mL)*	*Final volume before gelling (mL)*	*Surface area of scaffold (cm*^2^*)*	*Diameter of scaffold (cm)*	*Thickness of scaffold (μm)*
2	1.3	1.9	1.56	158 (SD 23.15)
2	0.25	0.32	0.64	—
1	1.3	1.9	1.56	103.6 (SD 24.74)

**FIG. 2.**
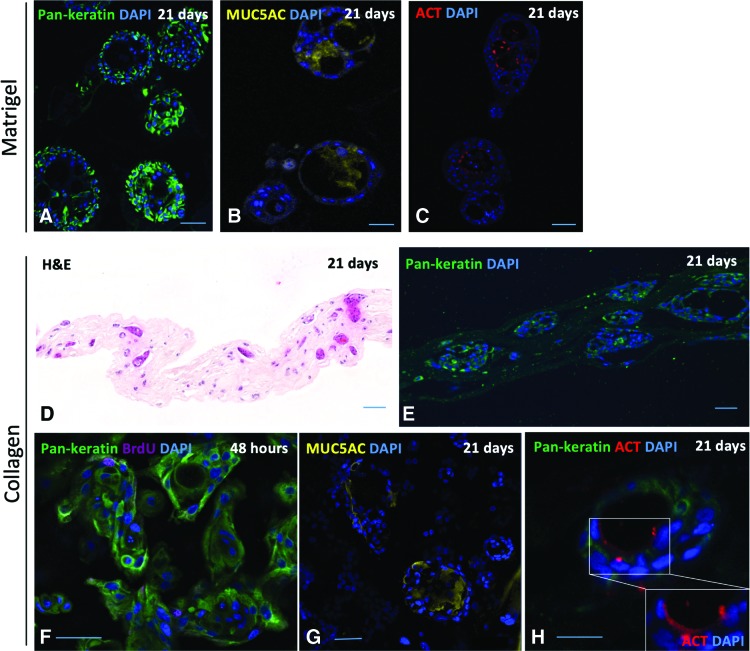
Three-dimensional culture of primary human airway basal cells in Matrigel and collagen I-based scaffolds. **(A)** Pan-keratin staining (*green*) of 3D tracheospheres generated using cultured human airway basal cells. **(B)** MUC5AC staining (*yellow*) of 3D tracheospheres. **(C)** Acetylated tubulin (ACT) staining (*red*) of 3D tracheospheres. Tracheosphere experiments were performed using cultured basal cells from one donor in technical triplicates. **(D)** H&E staining of cell-containing collagen I-based scaffolds after 21 days of culture. **(E)** Pan-keratin staining (*green*) of cell-containing collagen I-based scaffolds after 21 days of culture. **(F)** After 48 h in collagen I-based scaffolds, pan-keratin-positive cells (*green*) label with bromodeoxyuridine (BrdU; *purple*) indicating ongoing proliferation. **(G)** MUC5AC staining (*yellow*) of cell-containing collagen I-based scaffolds after 21 days of culture. **(H)** ACT staining (*red*) of cell-containing collagen I-based scaffolds after 21 days of culture. Collagen I-based scaffolds were produced using cultured basal cells from two independent donors in technical triplicates. DAPI was used as a nuclear counterstain for all immunofluorescence staining (*blue*). Scale bars = 50 μm. 3D, three dimensional; H&E, haematoxylin and eosin; DAPI, 4′,6-diamidino-2-phenylindole. Color images are available online.

**FIG. 3.**
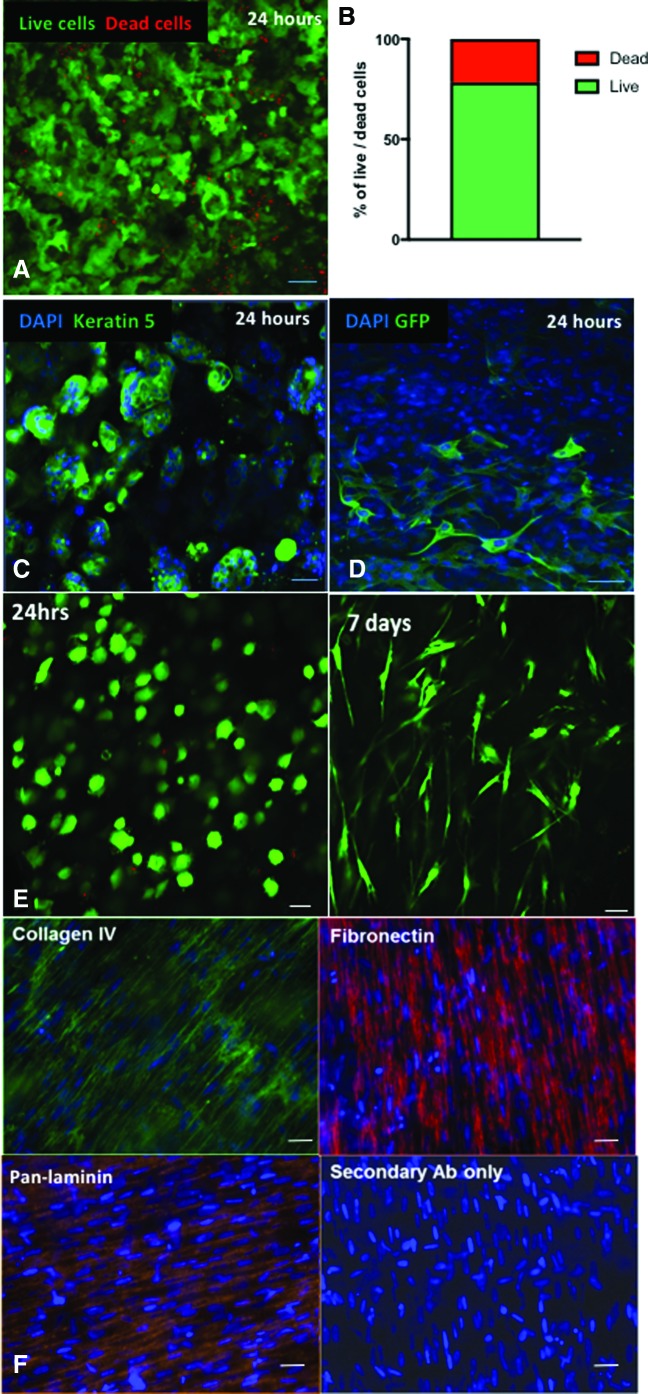
Generation of airway basal cell and lung fibroblast-containing collagen scaffolds for transplantation. **(A)** Top-down live/dead immunofluorescence staining of primary human epithelial cells in a collagen I scaffold 24 h after seeding demonstrates predominantly live cells (*green*) with sparse dead cells (*red*). **(B)** Cell counts of live and dead primary human epithelial cells embedded within collagen I scaffolds without fibroblasts. Data presented are a mean value from two independent scaffolds. **(C)** Top-down immunofluorescence staining of collagen I-embedded epithelial cells at 24 h demonstrates that they retain expression of the airway basal cell marker keratin 5 (*green*). Representative of three epithelial cell cultures from independent donors performed in at least technical triplicates. **(D)** When green fluorescent protein-labeled primary human lung fibroblasts are simultaneously seeded with primary human airway basal cells in collagen I scaffolds, fibroblasts (*green*) are observed throughout the scaffold. Representative of three independent scaffolds. **(E)** At 24 h and 7 days, scaffolds containing fibroblasts only were treated with calcein AM (Life Technologies, Cambridge) and imaged using whole-mount top-down confocal microscopy. Live fibroblasts were seen throughout the scaffold and had taken on a mature spindle-like morphology after 1 week of culture. **(F)** Following culture of fibroblasts within collagen scaffolds for 7 days, the scaffolds were fixed and stained for collagen IV, fibronectin, and mixed laminins. DAPI was used as a nuclear counterstain for all immunofluorescence staining. Scale bars = 50 μm. Color images are available online.

**FIG. 4.**
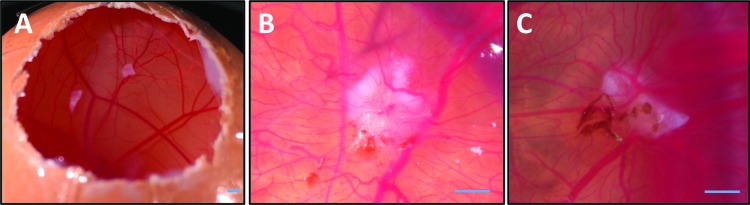
Vascularization of scaffolds on a chick chorioallantoic membrane assay. **(A)** Three eggs were windowed on embryonic day 8 for implantation of scaffolds. **(B)** On embryonic day 14, small blood vessels were seen to have integrated into the collagen scaffold, indicating neovascularization. **(C)** Decellularized dermis positive control scaffolds showed similar results. Scale bars = 2 mm. Color images are available online.

**FIG. 5.**
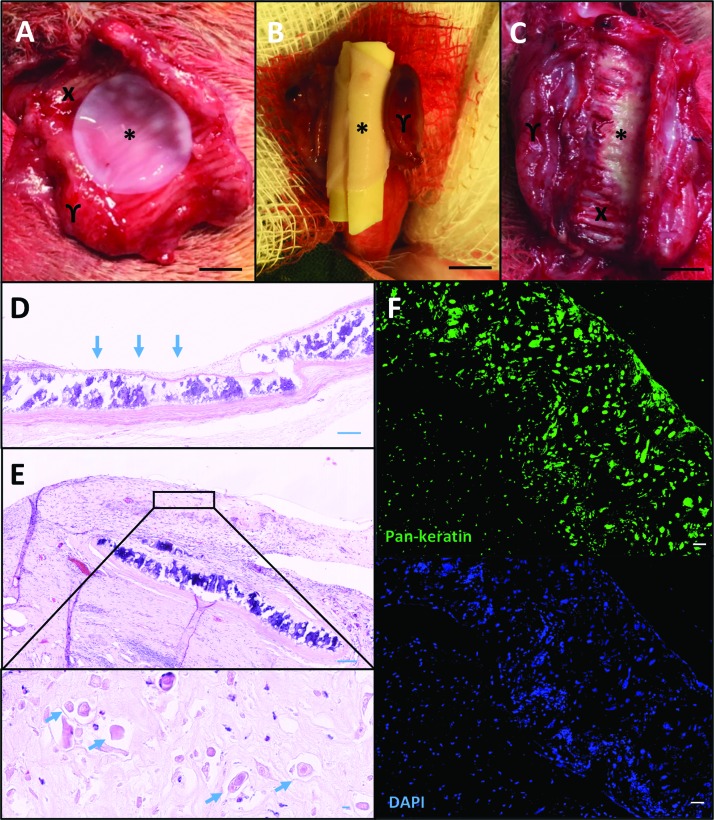
Engraftment of collagen-embedded epithelial cells *in vivo*. **(A)** Epithelial cell-containing collagen I-based scaffolds (^*^) were transplanted onto a section of prevascularized decellularized rabbit trachea (X) wrapped in a muscle flap (γ). **(B)** The graft was wrapped around a silicone stent. **(C)** After 1 week, the trachea was reopened and a high-definition digital photograph was taken of the luminal surface, which showed the integrated collagen-embedded epithelial graft (^*^). **(D)** H&E staining of formalin-fixed, paraffin-embedded trachea following revascularization in a muscle flap is shown. The exposed cartilage (*arrows*) is the site on which the collagen scaffold is grafted. **(E)** H&E staining of formalin-fixed, paraffin-embedded trachea showed the collagen graft (*solid line*) had integrated and epithelial structures were observed throughout the scaffold (*arrows*). **(F)** Immunofluorescence staining for the epithelial cell marker pan-keratin (*green*) and DAPI (*blue*). Scale bars = 5 mm **(A**–**C)** and 50 μm **(D**–**F)**. Color images are available online.

For the experiments described, a starting collagen concentration of 2 mg/mL was used. Two different sized scaffolds were prepared by adjusting the final volume before gelling. Scaffolds with a surface area of 0.32 cm^2^ were used in the experiments reported in Figures 2–4. Larger scaffolds with a surface area of 1.56 cm^2^ were used in the experiments reported in Figure 5. Nine readings were made at different locations on each of three independent scaffolds.

SD, standard deviation.

### Characterization of collagen I-based scaffolds containing human airway basal cells and lung fibroblasts

To develop a collagen scaffold-based method to protect airway basal cells from mechanical shear during transplantation, we first compared Matrigel to a collagen I matrix *in vitro*, as it is widely expressed in the lungs^27^ and is a more clinically relevant matrix. In a 3D tracheosphere assay,^19,23^ basal cells embedded in Matrigel formed spheroids that underwent lumen formation and demonstrated polarization and differentiation of luminal cells toward mature airway lineages, as demonstrated by the presence of MUC5AC+ mucosecretory cells and acetylated tubulin (ACT)+ ciliated cells (Fig. 2A–C). In parallel, we generated collagen I-based sheets containing airway basal cells and human lung fibroblasts by first producing a cell suspension in collagen I and then dehydrating it using RAFT absorbers. We maintained cells for 21 days *in vitro* and found that 3D spheroids were found throughout the collagen I matrix (Fig. 2D, E). Epithelial cells formed 3D spheroids containing BrdU+ proliferating cells within 48 h (Fig. 2F), consistent with previous work suggesting that spheroids are formed by a combination of cell aggregation and proliferation at this seeding density.^19^ Tracheospheres in Matrigel are known to be proliferative at this time point.^19,23^ We found spheroids that had undergone lumen formation and contained MUC5AC+ mucosecretory cells and ACT+ ciliated cells (Fig. 2G, H), despite maintenance in epithelial growth medium rather than airway differentiation medium for the initial 7 days of culture. Based on these data, we used scaffolds within 24 h of construction in our subsequent experiments such that basal stem/progenitor cells, rather than their differentiated progeny, are delivered.

To assess epithelial cell viability in the scaffolds, we performed a live/dead assay at 24 h in scaffolds containing only epithelial cells. A mean of 78% of epithelial cells survived the embedding and dehydration protocol used for scaffold generation (Fig. 3A, B). Embedded epithelial cells expressed the basal cell-associated protein keratin 5 (KRT5; Fig. 3C). To investigate the distribution of lung fibroblasts in these grafts, we embedded green fluorescent protein-expressing primary human lung fibroblasts and found that these were distributed throughout the scaffold (Fig. 3D).

To investigate the fibroblast growth pattern within collagen scaffolds, constructs containing only primary human lung fibroblasts were prepared and imaged using the cell viability marker Calcein AM (Life Technologies, Cambridge) at 24 h and 7 days. At 24 h, immature viable fibroblasts were seen throughout the scaffold (Fig. 3E). By 7 days, fibroblasts with a mature spindle-like morphology were seen throughout the scaffolds (Fig. 3E). At the same time point, extracellular matrix (ECM) production by fibroblasts was investigated by immunofluorescence staining for collagen IV, fibronectin, and pan-laminin. Evidence for the production of each of these proteins was found (Fig. 3F).

### Engraftment and vascularization of a collagen I-based scaffold in the CAM assay

The clinical application of grafts is reliant on effective engraftment at the donor site and, in particular, the biomaterial must support the ingrowth of host vascular tissue. While the ability of collagen I-based scaffolds to support vascularization has been demonstrated previously,^28^ we performed chick CAM assays in preparation for further *in vivo* applications. Acellular collagen I scaffolds were grafted onto the surface of three different CAM surfaces on embryonic day 8 (E8; Fig. 4A). Six days after grafting, CAM vessels were seen to penetrate the scaffold and had migrated over the surface (Fig. 4B). The extent of revascularization was comparable to decellularized dermis controls (Fig. 4C), a graft with established biocompatibility and which is used clinically for skin grafting.

### Engraftment of a collagen I-based graft onto decellularized trachea in an *in vivo* rabbit model

Satisfied that collagen I-based scaffolds were biocompatible in the CAM assay, we sought to apply airway basal cell and lung fibroblast-containing scaffolds *in vivo*. In light of the poor performance of acellular, nonvascularized scaffolds *in vivo*,^29^ we have developed an immunosuppressed rabbit model in which decellularized rabbit trachea is revascularized in preparation for a two-stage tracheal transplantation procedure. In this study, we revascularized rabbit tracheal scaffolds for 4 weeks before application of cell-containing collagen I scaffolds constructed 24 h previously (Fig. 5A). A stent was placed over the surface of the graft (Fig. 5B) to maintain patency and mimic a likely clinical scenario.^30^ The trachea was closed by suturing and remained in the neck of the immunosuppressed rabbit for 1 week. After retrieval and stent removal, the collagen I-based scaffold appeared integrated with the revascularized tracheal scaffold (Fig. 5C). Before grafting of the epithelial collagen scaffold, H&E staining demonstrated the exposed cartilage rings of the implanted trachea (Fig. 5D). Seven days after scaffold engraftment onto the implanted cartilage, H&E staining and immunofluorescence demonstrated the presence of keratin-positive epithelial cells throughout (Fig. 5E, F).

## Discussion

Our initial report of an airway basal cell- and lung fibroblast-containing collagen I-based scaffold suggests that it is biocompatible, is capable of vascularization, and can support basal cells, which maintain their capacity to proliferate and differentiate appropriately. Furthermore, the scaffold withstands engraftment onto a section of revascularized tracheal matrix, despite the use of a stent. With further development, this approach may be advantageous over more conventional forms of bioengineered epithelial grafts, such as reconstituted oral mucosa,^31^ where the epithelial layer is exposed on the surface and is likely to shear at the point of implantation due to surgical handling and by the friction of the overlying stent required to retain the graft within the airway. This scaffold might also help to support cell survival during the relatively hypoxic phase immediately after implantation, which is particularly important within the trachea, where large vascular channels are only found at the intercartilaginous junctions, making the graft bed less vascular when compared to the dermis of the skin.^32,33^

The method described in this study to generate collagen I-based scaffolds from a cell-containing gel can be modified to generate grafts of specific sizes or shapes. This is a clear advantage over other biological scaffolds such as decellularized dermis or small intestinal submucosa, which rely on an allogeneic donor supply and vary in thickness.^34^ In addition, these scaffolds might in future be modified to more closely mimic the airway ECM^35^ or to include factors that either support epithelial cell viability, such as a RHO-associated protein kinase inhibitor,^36^ or reduce the probability of basal cell differentiation, such as transforming growth factor β pathway inhibitors.^37,38^ The inclusion of proangiogenic factors such as vascular endothelial growth factor on the basal layer of the graft using a collagen layering technique,^39^ may also enhance revascularization of the scaffold.

The fate of the engrafted epithelial cells remains uncertain in this model and further studies using optimized airway basal cell culture conditions^40^ and/or donor-matched epithelial cells and fibroblasts would be beneficial. It is possible that epithelial stem/progenitor cells will migrate and repopulate the graft bed as the collagen resorbs with eventual differentiation and restoration of the epithelial layer, as has been possible using cultured epidermal stem cells grafted onto a prepared wound bed.^9,41^ This is supported by our demonstration that airway epithelial cells retain their capacity to differentiate and mature following collagen embedding *in vitro* (Fig. 2). Even if this is not the case, the successful delivery of living cells to a segment of airway devoid of mucosa is significant: despite apparent disorganization of epithelium applied using this method, it may be capable of acting as a biological dressing by promoting a productive local immune response to prevent infection and/or stimulating the ingrowth of native epithelium from surrounding tissue. This concept has been used clinically, where cadaveric dermis grafts are deployed to protect wound beds from infection and to help promote skin regeneration underneath the graft.^42,43^
